# What do we know about alcohol internet interventions aimed at employees?—A scoping review

**DOI:** 10.3389/fpubh.2023.929782

**Published:** 2023-01-26

**Authors:** Christopher Sundström, David Forsström, Anne H. Berman, Zarnie Khadjesari, Kristina Sundqvist

**Affiliations:** ^1^Department of Psychology, Stockholm University, Stockholm, Sweden; ^2^Centre for Psychiatry Research, Department of Clinical Neuroscience, Karolinska Institutet and Stockholm Health Care Services, Stockholm, Sweden; ^3^Department of Psychology, Uppsala University, Uppsala, Sweden; ^4^Behavioral and Implementation Science Research Group, School of Health Sciences, University of East Anglia, Norwich, United Kingdom

**Keywords:** alcohol, digital intervention, internet intervention, workplace, employee, scoping review

## Abstract

**Background:**

Internet interventions are a promising avenue for delivering alcohol prevention to employees. The objective of this scoping review was to map all research on alcohol internet interventions aimed at employees regardless of design, to gain an overview of current evidence and identify potential knowledge gaps.

**Methods:**

We conducted a literature search in three data bases (PsycInfo, PubMed, and Web of Science). Eligibility criteria were that (1) the study targeted employees age ≥18 years; (2) the intervention was delivered predominantly online; (3) the study focused specifically or in part on alcohol use; and (4) the study was published in English in a peer-reviewed academic journal. Two reviewers independently screened, reviewed, and extracted data.

**Results:**

Twenty studies were included, of which 10 were randomized controlled trials, five were secondary analyses, three were feasibility trials, one was a cohort study and one described the rationale and development of an intervention. No qualitative studies were found. Randomized trials tended to show effects when interventions were compared to waitlists but not when more intensive interventions were compared to less intensive ones. We identified two design-related aspects where studies differed; (1) whether all applicants were included regardless of alcohol use level and (2) whether the intervention was explicitly framed as alcohol-focused or not. Significant recruitment problems were noted in several studies.

**Conclusions:**

Alcohol internet interventions hold promise in delivering alcohol prevention to employees, but heterogeneity in study design and difficulties in recruitment complicate interpretation of findings.

**Systematic review registration:**

https://osf.io/25x7e/, Open Science Framework.

## 1. Introduction

Alcohol remains a major risk factor for burden of disease in most parts of the world (1), but only one in six receive treatment (2). Since the majority of adults are employed and people spend most of their time at work, the workplace has been regarded as a valuable setting for provision of alcohol prevention (3, 4). Aside from the potential benefit for employees suffering from alcohol misuse, such as access to free and confidential evidence-based help, alcohol prevention is likely of great value for employers as the cost of alcohol problems in the workplace, measured in both absenteeism (being away from work due to consequences of alcohol use), presenteeism (being less productive at work due to consequences of alcohol use) and accidents, is substantial (5, 6). In recent decades, implementation of alcohol prevention programs in the workplace has therefore become increasingly common (3), complementing general workplace policies (7).

Despite the appropriateness and potential of alcohol prevention aimed at employees, there is a general lack of research in the area, with systematic reviews identifying few randomized controlled trials (8, 9). The lack of research may partly be attributed to the challenging context; workplace demands may be prioritized over research projects, insufficient support from the organization and from managers of the research project may create resistance among employees, and staff turnover and retirement may have negative consequences for follow-up. Aside from these issues common to all research conducted in the workplace, research on alcohol prevention introduces an additional challenge; there may be a significant fear of disclosing alcohol problems to one's employer, as this may negatively impact one's work situation.

A major development in mental health research in recent decades is the area of internet interventions, i.e., therapeutic interventions delivered *via* the internet. These interventions range widely in content from full cognitive behavior therapy treatment manuals delivered on internet platforms over weeks or months, to smartphone applications consisting of brief tips and techniques to address the problem in question “just-in-time.” They can also be delivered in blended format (i.e., face-to-face and internet combined), as well as with or without written guidance from a coach or health care professional (10). By now, a large body of evidence shows internet interventions to be effective in helping people with a range of mental health problems (11) and, although most of this evidence stems from research in community samples, these interventions are increasingly being integrated with routine mental health care (12). Internet interventions targeting both mental health (i.e., stress, depression, and alcohol) and general health (i.e., obesity, cardiovascular disease, and sedentary behavior) have been investigated specifically in the workplace (13), and two systematic reviews have attempted to summarize the evidence; one an analysis of 22 randomized controlled trials (RCTs) (14), and the second an analysis of 50 RCTs including a series of focused meta-analyses (15). The first of these, the systematic review by Howarth et al. (14), concluded that interventions targeting specific health behaviors such as sedentary behavior, insomnia, and heart health were moderately effective, while studies targeting more complex health behaviors such as alcohol problems and smoking, were less effective. Results from the six included studies on alcohol internet interventions were mixed, with significant findings only related to different subgroups. The second of these, the systematic review with a series of meta-analyses conducted by Philips et al. (15), showed moderate effects for interventions aimed at stress, insomnia and burnout, and lower effects for interventions aimed at depression and anxiety. The effects were non-significant for alcohol consumption, but only two studies were included in this meta-analysis. Further, in the only individual patient data meta-analysis (IPDMA) on alcohol internet interventions, a sub-group analysis of settings (community, healthcare, and workplace) was performed. Interventions conducted in community and healthcare settings were found effective in reducing alcohol consumption, while findings on interventions conducted in workplace settings were inconclusive. Of note, only three RCTs conducted in the workplace were included in the IPDMA, compared with seven in healthcare settings and 17 in community settings (16).

The aforementioned reviews were restricted to RCTs. However, focusing solely on RCTs may provide limited information on the current evidence in a setting such as the workplace where controlled trials are evidently rare and complicated to conduct (9). As there have been no previous attempts to summarize the wider literature on alcohol internet interventions in the workplace, a broad approach looking at all available research regardless of design, would be most likely to help the field advance at this stage. In order to gain an overview of the field and identify knowledge gaps, we therefore conducted a scoping review of all available evidence (17). We define alcohol internet interventions as any primary, secondary, tertiary prevention or treatment program designed to reduce alcohol consumption, delivered through the internet. Specifically, we were interested in mapping the characteristics of published research on internet alcohol interventions aimed at employees, asking the following specific questions:

1. What study designs were used?
2. What were the characteristics of the workplace setting where the studies were conducted?
3. How were employees approached?
4. What types of interventions were used?
5. What were the demographics of participants?
6. What were the main findings?

## 2. Materials and methods

### 2.1. Study design

This study was a scoping review attempting to summarize all published literature on internet alcohol interventions aimed at employees, using established methodology (17). All reporting was conducted in accordance with the PRISMA extension for Scoping Reviews (18).

### 2.2. Eligibility criteria

As this was a scoping review, we did not focus solely on RCTs and other quantitative evaluations but rather included all published research investigating this topic.

In order to be included in the review, the following eligibility criteria had to be met:

1. The study targeted employees ≥18 years old.
2. The intervention was predominantly provided over the internet (including mobile apps).
3. The study focused specifically or in part on alcohol use.
4. The publication was published in English in a peer-reviewed academic journal from January 2000 onwards (considering the fact that internet interventions were rarely investigated before this date).

Reviews and protocols were not included. In some cases, interventions were simultaneously aimed at several health-related behaviors (i.e., alcohol, physical activity, smoking etc). We then included only those studies where an alcohol-related outcome was reported.

### 2.3. Literature search strategy

A systematic literature search was conducted, with relevant articles published between January 2000 and February 2021 retrieved from the electronic databases PsycInfo, PubMed, and Web of Science. We combined search terms for “internet,” “alcohol problems,” and “employee” to search titles, keywords, or abstracts. Our search terms in PsycInfo were: (((work^*^ or corporate or white-collar or professional or labor or labor or industrial or staff or organization or organization or employ^*^) **and** (CBT or cognitive behavio^*^ therapy or treatment or brief intervention or intervention) **and** (Internet or web or online or compute^*^ or cybertherapy or digital or e-intervention or e-mental health or e-therapy or eHealth or e-Health or tele-therapy or telehealth or app) **and** (drinking or alcohol))).ab,kw,ti (see Appendix for full search strategy in other databases). Further, reference lists of included studies and previously published reviews on internet interventions and workplace interventions were hand-searched to identify additional eligible studies. In December 2021, we updated the search to identify any studies that had been published since our initial search in February 2021. We made no searches for gray literature.

### 2.4. Data collection, extraction, and synthesis

Once the literature search had been conducted, all duplicates (studies found in more than one database) were removed. Articles were then independently screened in Excel sheets in three stages (title/abstract/full text) by authors CS and DF. At each stage, articles that clearly failed to meet eligibility criteria were excluded. If during the title and abstract screening stage, either of the two screening authors was unsure of whether or not a study should be excluded, that study was retained to be reconsidered in the next stage of screening. Any uncertainty or disagreement concerning whether a study should be excluded during the full text stage of screening was resolved in discussion with author ZK. After the screening process, authors CS and DF extracted relevant data from each eligible article: study characteristics, intervention characteristics and outcome data. Research was then summarized narratively and inductively with a primary focus on responding to the research questions and identifying research gaps.

## 3. Results

**Figure 1:**
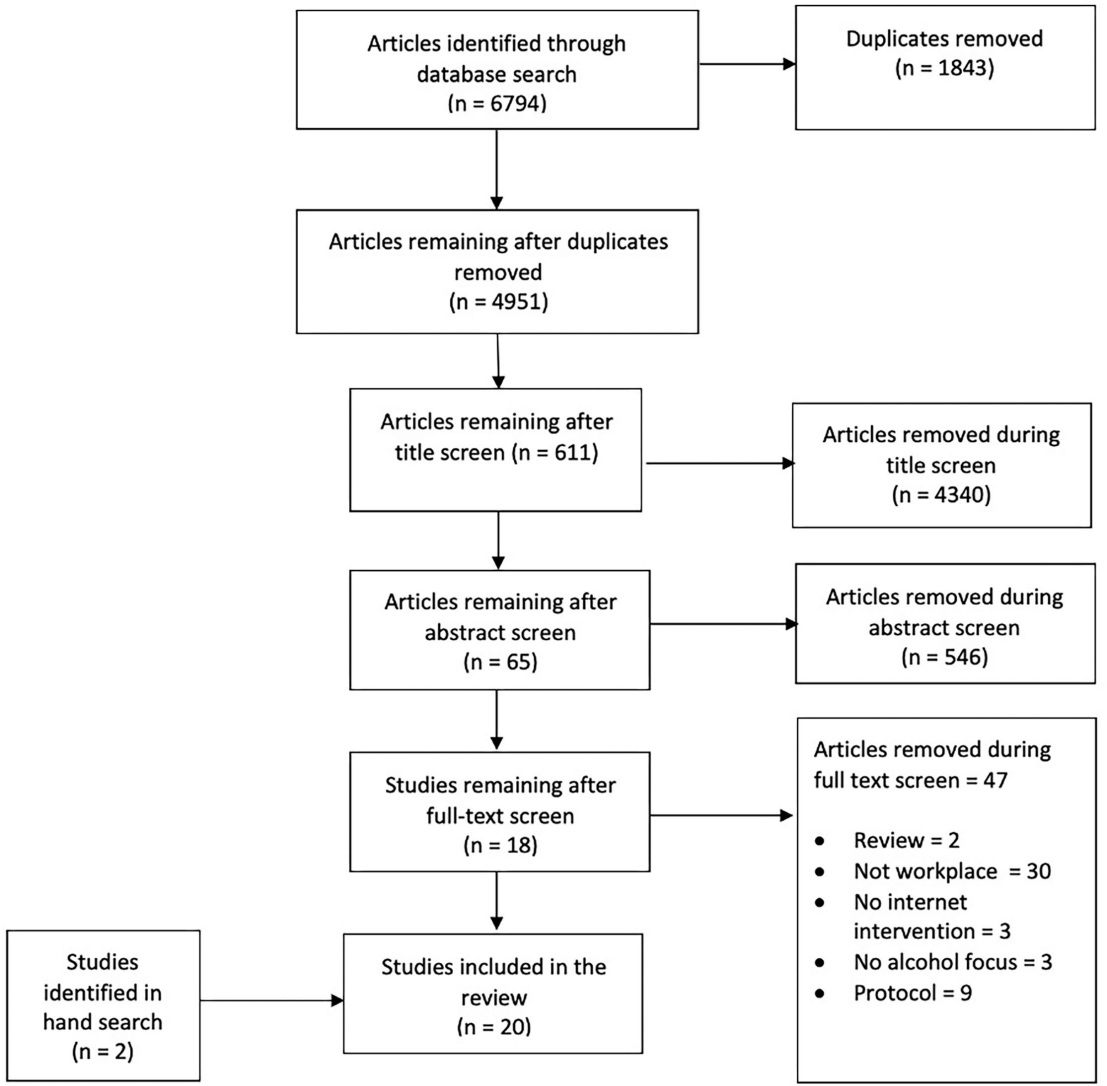
Flow of studies through the selection process.

The combined initial literature search yielded 6,794 articles. Of these, 1,843 were removed as they were duplicates, leaving 4,951 articles for the title screen after which 611 remained. After the abstract screen, 65 remained, and after the full text screen, a total of 18 articles were included in the review. Further, two studies were identified in a hand search, leaving a total of 20 studies. See Figure 1 for a flowchart. See Table 1 for a presentation of the studies included.

**Table 1:** Extraction data from the included studies.

**References, country**	**Study design**	**Study focus**	**Workplace**	***n* (% female)**	**Age (M, SD)**	**Name and therapeutic approach of the internet intervention**	**Alcohol-related and work-related outcomes**	**Findings**	**Comments**
Araki et al. (19), Japan	RCT with three groups: 1. Face-to-face 2. Internet (e-mail) 3.Control	Alcohol	Manufacturing plant	36 (0%)	43.8 (7.5)	No name of intervention reported Therapeutic approach: alcohol education (a series of emails)	Drinks in preceding week Alcohol knowledge Attitude toward change GTP (blood test indicating hepatic dysfunction)	The face-to-face intervention was significantly more effective than both the internet intervention (e-mails) and the control group in: •decreasing drinks in preceding week (*F* = 2.33 = 3.35, *p* = 0.0472) •increasing alcohol knowledge (*F* = 2.33 = 7.72, *p* = 0.0018) •increasing positive attitude toward change (*F* = 2.33 = 4.79, *p* = 0.0149) There were no significant differences between groups in GTP (*F* = 2.33 = 2.47, *p* = 0.0999) There were no significant differences between the internet intervention and the control group	The reason that the face-to-face intervention was more effective may be because the investigator providing the intervention was able to provide tailored feedback, in contrast to the email intervention which was standardized
Billings et al. (20), US	RCT with two groups: 1. Internet 2. Waitlist	Alcohol and stress	Technology company	309 (71%)	72% were in the 30–49 age range; no mean (SD) reported	“Stress and mood *Management”* Therapeutic approach: cognitive behavior therapy	Stage of change Work productivity	Those in the internet intervention: •showed a significantly more positive transition in their binge drinking stage of change (*F* = 7.57, *p* < 0.01) •displayed a significantly greater reduction in stress (*F* = 5.23, *p* < 0.05) •showed a trend toward an increase in work productivity (*F* = 3.85, *p* < 0.10)	Authors speculate that the intervention focus on stress makes the program less stigmatizing. Stigmatized behavioral health problems may thus be successfully addressed by embedding prevention material into the stress management framework
Boss et al. (21), Germany	RCT with three groups: 1. Guided internet intervention 2. Unguided internet intervention 3. Wait list	Alcohol	Workplace sectors: •Service: 23% •Economy: 17% •Health: 11% •Social: 9% •Information technologies: 6% •Other: 33%	434 (59%)	47.5 (9.8)	“*Be Smart—Drink Less”* Therapeutic approach: cognitive behavior therapy with personalized normative feedback	Drinks in preceding week Emotional irritation Cognitive irritation	When combined, the two intervention groups, compared to the waitlist group, showed significantly larger reductions in drinking 6 weeks after baseline (*B* = −4.85, CI: −7.02 to −2.68, *p* < 0.001, effect size = 0.30), as well as in depression, anxiety, stress as well as work-related outcomes in terms of emotional and cognitive irritation (effect sizes ranging from 0.09 to 0.36) There were no significant differences between the guided and unguided group	There were major challenges in recruitment. Authors state that it might be beneficial to collaborate closer with occupational health practitioners and other existing services in order to recruit successfully
Brendryen et al. (22), Norway	RCT with two groups: 1. intensive internet intervention 2. e-booklet	Alcohol	Four organizations •Public hospitals •A county •Municipality dept •Consulting company	85 (52%)	43 (11)	“*Balance”* Therapeutic approach: cognitive behavior therapy	Drinks in preceding week	At 2 and 6 months, there were no significant differences between the groups when using a baseline observation carried forward approach (2 months: *B* = 2.96, 95% CI = −0.50 to 6.42, *p* = 0.09, 6 months: *B* = 1–07, 95% CI = −1.29 to 3.44, *p* = 0.37).	The recruitment potential was overestimated, and the trial was terminated before reaching the required number of participants as defined by the power analysis.
Buntrock et al. (23), Germany	Secondary analysis of Boss et al. (21) Economic evaluation of guided and unguided internet-based interventions compared with a wait-list	Alcohol	See Boss et al. (21)	See Boss et al. (21)	See Boss et al. (21)	See Boss et al. (21)	Costs from a societal perspective Costs from an employer perspective	Cost-effectiveness from a societal perspective: the guided intervention had a probability of 55 and 54% respectively of being the most efficient strategy at a willingness-to-pay (WTP) of €0, compared with the unguided intervention and wait-list. At a WTP of €20,000 per QALY gained, the probability was 78% Cost-effectiveness from an employer perspective: the guided intervention had a higher probability of positive return on investment (81%) compared with the unguided intervention (58%)	This is the only study that investigates cost-effectiveness of internet alcohol interventions among employees The study suggests that guided interventions may be more cost-effective than unguided ones
Colkesen et a. (24), Netherlands	Cohort study	Health behaviors related to the development of cardiovascular disease	Seven companies, mainly highly educated (i.e., white-collar workers)	2,289 (39%)	63% were in the 30–49 range; no *M*/SD reported	*The Prevention Compass* Therapeutic approach: personalized normative feedback	Initiation of health behavior change (alcohol intake and other behaviors)	Among those receiving alcohol-related feedback (*n* = 262), 64 (24%) reported having reduced alcohol intake 4 weeks after having received the intervention. More highly educated employees were less likely to reduce alcohol intake (OR 0.50, 95% CI 0.25–0.99). Among those who received feedback on diet and physical activity, change initiation was more common (38 and 44% respectively), while change initiation in smoking was less common (3%)	Attrition was high; 6,790 employees were invited. Of these 34% (2,289) participated. Of these, only 28% (638) responded to the 4-week follow-up
Doumas and Hannah (25), US	RCT with three groups: 1. Internet intervention 2. Internet intervention + motivational Interviewing 3. Control group	Alcohol	Five local companies	124 (73%)	All participants were between 18–24; no mean/SD reported	*Check your drinking* Therapeutic approach: personalized normative feedback	Drinking quantity Peak consumption Frequency of drinking till intoxication At 30-day follow-up	When combined, the two intervention groups showed significantly larger reductions compared to the control group in weekend drinking [*F*_(1,117)_ = 9.10, *p* < 0.01], peak consumption [*F*_(1,117)_ = 4.59, *p* = < 0.05] and frequency of drinking till intoxication [*F*_(1,117)_ = 4.67, *p* < 0.05]. No difference was identified between the two active groups	46% of eligible participants participated in the study, and of these 63% participated in the follow-up
Hamamura et al. (26), Japan	Feasibility, non-randomized to two groups: 1. Mobile app 2. Assessment only	Alcohol and stress	Recruitment took place *via* a research marketing company	557 (41%)	39 (9.6)	“*Self Record”* Therapeutic approach: cognitive behavior therapy	Daily drinking questionnaire	In the intervention group, there were significant increases in typical drinking (*p* = 0.001) and heavy drinking (*p* = 0.01) as compared to the assessment-only group, in the intention-to-treat analyses	Those interested in using the app received the intervention, those not interested received the control. This might explain the negative findings of those receiving the intervention
Khadjesari et al. (27), UK	RCT with two groups: 1. Normative feedback 2. Assessment only	Alcohol	A large unnamed organization	1,330 (25%)	48 (no SD provided)	*Healthy Choices Online* Therapeutic approach: personalized normative feedback	Number of drinks in preceding week AUDIT EQ-5D (quality of life)	No significant differences between groups were found on any of the outcomes; number of drinks (95% CI −4.7 to 16.9%, *p* = 0.30), AUDIT (95% CI = −4.3 to 4.5%, *p* = 1.0), EQ-5D (95% CI = −2.0 to 1.7%, *p* = 1.0)	3% of the employees in the organization participated. It was not clear whether participants actually read the alcohol feedback, particularly as it was presented alongside feedback on other health behaviors
Khadjesari et al. (28), UK	Feasibility study, with a focus on participation and attrition	Alcohol	Six organizations •three local authorities •one hospital •one university •one private sector organization	1,254 (65%)	43 (11)	See Khadjesari et al. (27)	Questionnaire on how the intervention was perceived	An average of 8% were recruited from the participating organizations. 25% were drinking at hazardous levels 64% completed 3-month follow-ups, and 95% of these were supportive of workplaces offering employees a “health check” Those with a higher AUDIT score were more likely to be concerned about their employer seeing the results (22 vs. 13%, OR 2.12 95% CI: 1.36, 3.30)	A challenge to delivering internet interventions in the workplace is to attract those most likely to benefit; If completion is made compulsory, this may have implications on the veracity of the reported data
Kouwenhoven-Pasmooij et al. (29), Netherlands	RCT with two groups: 1. Extensive intervention (Internet intervention with 7 coaching sessions) 2. Limited intervention (Internet intervention only)	Health behaviors	Three organizations; •the military •the police •academic hospital	491 (19%)	50.9 (5.8)	*Perfect Fit* Therapeutic approach: personalized normative feedback	Number of drinks per week on a 7-point scale	No differences found between the groups, but an overall within-group decline in excessive alcohol use was observed	The authors speculate that feedback from the intervention may have acted as a warning that subjects needed to improve rapidly, in particular in the military and the police where good physical fitness is a prerequisite for the job. This may explain the finding that improvements were seen in both intervention groups
Matano et al. (30), US	Study describing the rationale and development of an intervention	Alcohol and stress	One major Silicone Valley work site	No data on employees using the intervention are presented	No data on employees using the intervention are presented	*Coping Matters* Therapeutic approach: cognitive behavior therapy + personalized normative feedback	No data on employees using the intervention are presented	No data on employees using the intervention are presented	This paper argues that an easily accessible internet intervention is needed in the workplace. The intervention in question is completely anonymous and is described as simultaneously providing primary and secondary prevention
Matano et al. (31), US	RCT with two groups: 1. Full individualized feedback 2. Limited individualized feedback (no feedback on alcohol) Participants were classified as medium- and low-risk drinkers based on AUDIT and CAGE scores and then randomized. Those classified as high-risk drinkers were not included for ethical reasons	Alcohol and stress	One major Silicone Valley work site	145 (78%)	39.9 (11.3)	See Matano et al. (30)	Reported alcohol consumption over the past 3 months (quantity and frequency of type of drink, i.e., wine, beer etc)	Medium-risk drinkers: those receiving the full individualized feedback reported a significant decrease in beer binges (*p* = 0.01) Low-risk drinkers: those receiving the full individualized feedback reported: •A significant decrease in beer binges (*p* = 0.02) •A significant decrease in hard liquor binges (*p* = 0.05)	The sample size for the targeted group (medium-risk drinkers) was low. Difficulties in recruitment were reported, and may be due to concerns of confidentiality The authors state that the challenge for alcohol prevention at the workplace is to provide a screening tool that offers differential responses depending on the employee's drinking pattern
Mauriello et al. (32), US	Feasibility study	Alcohol	Seven worksites; a university, a research firm, an accounting firm, a wellness center and several restaurants	166 (69%)	38.8 (11.2)	*Responsible drinking* Therapeutic approach: personalized normative feedback and the transtheoretical model	Evaluation questionnaire	The post-intervention evaluation questionnaire revealed: •High satisfaction (98% stated that the program was easy to use) •Greater intention to reduce number of drinks [*t*_(152)_ = −6.90, *p* < 0.01] and number of days drinking (*t*_(152)_ = 6.46, *p* < 0.01) after the intervention	This is the first intervention that offers tailored feedback on Trans Theoretical Model constructs for each stage of change. When fully developed the intervention will be the first to offer both normative and ipsative feedback on TTM progression
Niessen et al. (33), Netherlands	Secondary analysis of Colkesen et al. (24); determinants of participation	Health behaviors	Five organizations: •Two banks •A financial institution •A university medical center •A technology corporation	2,686 (46%)	43.2 (9.5)	See Colkesen et al. (24)	Alcohol consumption scale 0–4	Those with higher alcohol consumption at baseline were more likely to participate in the study (*p* = 0.001)	There was no selective participation related to education, sex or age
Pemberton et al. (34), US	RCT with three groups:1. Drinker's Check-up 2. Alcohol Savvy 3.Waitlist	Alcohol	Eight workplaces—two each from the four military branches •Army •Navy •Air Force •Marine Corps	3,070 (17%)	69% were in the 21–34 range; no *M*/SD reported	1. Drinker's Check-up Therapeutic approach: personalized normative feedback 2. Alcohol Savvy Therapeutic approach: cognitive behavior therapy	Seven different alcohol measures regarding consumption in the last month: average number of days that alcohol was used, Average number of drinks consumed per drinking occasion and Number of days perceived drunk	At 1 month, Drinker's Check-up had several significant effects on alcohol-related outcomes as compared to waitlist (average days per drinking occasion *d* = 0.10, estimated peak BAC *d* = 0.16, frequent heavy episodic drinking status *d* = 0.11) but there were no significant differences between Alcohol Savvy and waitlist. There were no significant differences between intervention groups and waitlist at 6 months	There was considerable attrition (1 month = 66%, 6 month = 77%), which raises the question of response bias. However, the researchers attempted to control for this with sensitivity analyses
Solenhill et al. (35), Sweden	RCT with three groups: 1. Tailored internet health feedback + telephone health coaching 2. Tailored internet health feedback 3. Control group	Health behaviors	Companies in the transport industry	3,876 (33%)	43 (10.7)	*Lifestyle Health Intervention* Therapeutic approach: personalized normative feedback	Number of occasions of alcohol consumption in the past year	There were no significant differences between the groups	The intervention did not have a positive health effect, but an increased short-term motivation to change health behaviors related to diet and physical activity was observed (although this was not the case with alcohol)
Westrup et al. (36), US	Secondary analysis of Matano et al. (31) Satisfaction	Alcohol and stress	Unidentified worksite	187 (77%)	40.9 (11.5)	See Matano et al. (30)	Employee reactions to the website with a follow-up survey	The majority of participants (78%) only used the intervention on 1 day Those with high-risk drinking used the website significantly more often (*p* < 0.05) than those with moderate- or low-risk drinking, average web site minutes: high risk = 19.9 (SD = 13.3), moderate risk = 14.6 (SD = 10.8), low risk = 14.8 (SD = 10.7) 8% of participants reported a positive change in relation to alcohol at post-intervention follow-up.	Three-quarters of participants were female, which raises the question of whether internet interventions for problem drinking appeal more to women. Participants were mainly highly educated
Williams et al. (37), US	Secondary analysis of Pemberton et al. (34); mediating mechanisms	Alcohol	Eight workplaces—two each from the four military branches •Army •Navy •Air Force •Marine Corps	3.070 (gender distribution not presented)	Not presented	1. Drinker's Check-up Therapeutic approach: personalized normative feedback 2. Alcohol savvy Therapeutic approach: cognitive behavior therapy	Average number of days Average number of drinks Number of days perceived drunk Binge drinker status Binge drinking episodes Heavy drinker status Estimated peak BAC	A number of descriptive norms among those receiving Drinker's Check-up mediated frequency and quantity of drinking, which was not the case with the Alcohol Savvy program	There was considerable attrition
Williams (38), US	Secondary analysis of Pemberton et al. (34): differences in mediating mechanisms based on gender	Alcohol	Eight workplaces—two each from the four military branches •Army •Navy •Air force •Marine corp	2,384	28.2 (no SD reported)	Drinker's Check-up Therapeutic approach: personalized normative feedback	See Williams et al. (37)	Mediation by norms about the number of drinks peers consumed did not vary significantly by gender Mediation by norms about how often peers drank (i.e., number of drinking occasions) differed significantly by gender for the number of days alcohol was consumed (CI = 0.023–1.658), the number of binge episodes (CI = 0.008–0.720), heavy drinker status (0.014–0.494), and the number of drinks consumed per drinking episode (0.028–0.762)	The smaller number of females in the original study may account for the overall lack of effectiveness of norms about drinking frequency as a mechanism of behavior change since its impact was largely confined to women

### 3.1. Characteristics of included studies

The 20 articles reflected 16 unique studies and 13 projects; (see Table 1). Geographically, nine of the studies were conducted in the US, three in the Netherlands, with two studies conducted in the UK, Germany, and Japan respectively, and one each in Norway and Sweden. A total of 14,166 participants were included in the studies.

### 3.2. What study designs were used?

Of the 20 studies identified, 10 were randomized controlled trials (19–22, 25, 27, 29, 31, 34, 35), five were feasibility, non-randomized trials or cohort trials (24, 26, 28, 32, 36), four were secondary analyses of previously conducted randomized controlled trials or cohort studies (23, 33, 37, 38) and one was a paper describing the rationale and development of an intervention (30). No qualitative studies were identified.

### 3.3. What were the characteristics of the workplace setting where the studies were conducted?

Of the 20 included articles, 11 were conducted in several workplaces simultaneously (22, 24, 25, 28, 29, 32–35, 37, 38) and six were conducted in one single workplace (19, 20, 27, 30, 31, 36). Three studies were not formally conducted in a workplace but recruited employees through health insurance companies' websites and print membership magazines as well as in print newspaper articles (21, 23), or used a research marketing company to recruit participants who reported being employed (26).

There was large variation both between and within studies in terms of sectors. In the six articles reporting on studies conducted in a single workplace, information about sector was sparse; only two revealed the sector of the workplace where the study was being conducted (19, 20), three simply stated recruiting from “a large Silicone Valley work site” (30, 31, 36) and one stated recruiting from a “large UK-based organization” (27). Among the 12 articles reporting on studies recruiting participants from several different workplaces, information about sector was usually brief (see Table 1). For example, Brendryen et al. (22) recruited from both public hospitals, a county, the municipality, and a consulting company and Kouwenhoven-Pasmooij et al. (29) recruited from the military, the police and an academic hospital. Three articles reported on a study conducted solely in the military, albeit from four different branches (34, 37, 38). One study used a research marketing company to recruit participants and did not report information on the sectors in which the participants worked (26).

The most common way of informing employees about the study was through employee emails or web portals belonging to the workplace(s) where the study was being conducted (20, 22, 24, 27, 33, 35). In some studies, both emails and/or web portals, as well as more traditional methods such as magazines, posters, recruitment flyers and television advertisements were used (28, 29, 32, 34, 37, 38), while in other studies only newspapers, brochures and flyers were used (30, 31, 36). In one study, only employees with abnormal levels in their serum *y*-GTP (i.e., blood test indicating hepatic dysfunction) from a workplace health check were approached (19). As previously mentioned, in three articles, no workplace was included in the recruitment process (21, 23, 26). One study did not specify which recruitment method was used (25).

### 3.4. What types of interventions were used?

The content of the interventions in the studies fell into two main categories; personalized normative feedback (PNF) and cognitive behavior therapy (CBT). PNF is a form of brief intervention aiming to inform individuals about their consumption patterns in comparison to those in the general population (39), while CBT is a form of psychological treatment that focused on the interplay of behaviors and thoughts. It should be noted that these interventions blend into one another sometimes, with some PNF interventions including CBT strategies and drinking diaries, and some CBT interventions including a PNF component.

Most interventions included PNF. Some of these interventions offered PNF for alcohol only (25, 32, 34, 37, 38), while in others, the intervention provided PNF on a host of health behaviors such as smoking, fruit/vegetable intake and physical activity (24, 27–29, 33, 35). In two of these later studies, the explicit objective of the researchers was to investigate the effects of PNF on alcohol consumption, with other health-related behaviors included in the intervention only to mask the alcohol focus of the study (27, 28), while in the other studies, the explicit research question did not focus exclusively on alcohol but on effects of PNF on all health behaviors (24, 29, 35). In one study, the intervention consisted of alcohol education, seemingly without any PNF (19).

Of the interventions based on CBT, two had an explicit focus on alcohol consumption (21, 22), while three interventions were mainly framed as focusing on stress and how to avoid using alcohol when coping with stress (20, 26, 30).

Four studies included some form of human contact in one of the groups. In one study, the intervention was delivered together with optional guidance from a therapist over the treatment platform (21), in a second study the intervention was delivered with coaching sessions (29), in a third study with telephone calls (35) and in a fourth study with motivational interviewing sessions (25).

### 3.5. What were the demographics of participants?

There was variation between the studies, with some including a large majority of females (20, 25, 31) some including a majority of males (24, 27, 29, 34), and one study including men only (19). Pemberton et al. (34) included 17% females in the military and Solenhill et al. (35) included 33% females in the transport industry, whereas Doumas and Hannah et al. (25) included 73% females among young people working in local companies, and Billings et al. (20) included 71% females from a technology company. The mean age of participants in most studies was between 40 and 50 years, with the exception of one study which only included young adults between the ages of 18 and 24 (25). Profession, education level and socioeconomic status were rarely reported.

### 3.6. What were the main findings?

A host of different alcohol outcomes were used in the studies of which the most common one was number of standard drinks consumed or some similar calendar-based way of assessing recent consumption (21, 22, 25–27). Other forms of self-reported alcohol outcomes were also used, such as alcohol knowledge (19), stage of change (20) and initiation of alcohol-related change (24). Only one study included a biological outcome measure (19).

In terms of intervention effects in the RCTs, seven studies used wait-lists as control groups to assess effects. In four of these, the internet interventions were found more effective than the waitlist in at least one alcohol-related outcome (20, 21, 25, 34) while in three studies, no such differences were found (19, 27, 35). Three studies compared the internet intervention to an active control group: Brendryen et al. (22) compared the internet intervention to an e-booklet with alcohol education, Matano et al. (31) compared a more extensive internet intervention to a less extensive internet intervention and Pemberton et al. (34) compared two different internet interventions; one based on CBT and one based on PNF. None of these studies found significant differences between the internet intervention and the active control. One study was non-randomized, with those wishing to use the internet intervention (a mobile app) receiving it and those not interested receiving assessment only. In this study, those choosing the internet intervention reported a significant *increase* in typical drinking and heavy drinking compared to the assessment group (26). The four studies comparing an intervention with and without human contact, found no significant differences between the groups (21, 25, 29, 35). A cost-effectiveness analysis of one of these studies (21), however, found that the guided intervention was more cost-effective than then unguided intervention (23). In the only study comparing a human-delivered intervention with an internet intervention, the human-delivered intervention was found to be more effective (19).

Some studies also investigated mental health outcomes. Billings et al. (20) found that those receiving the internet intervention reduced their stress as well as increased their knowledge about anxiety and depression compared to the waitlist and Boss et al. (21) found that the internet intervention reduced stress, anxiety and depression compared to the waitlist. Khadjesari et al. (27) found no significant differences in quality of life between the intervention and the control group. Hamamura et al. (26) found that those using the internet intervention (mobile app) had an increase in anxiety. As mentioned, this study was non-randomized thereby greatly reducing internal validity.

Only two studies reported work-related outcomes: Billings et al. (20) found that users of the intervention improved their ability to handle time and scheduling demands of their job when compared to controls, and also that they improved the quantity and quality of completed work. Boss et al. (21) found significantly greater reductions in emotional and cognitive irritation, a way of assessing rumination in the workplace context (40).

One study described the rationale and development of an intervention, building the case for developing an alcohol internet intervention that can simultaneously offer primary and secondary prevention (30). Three studies were feasibility studies looking at recruitment potential and/or user satisfaction (27, 32, 36). Four studies presented secondary analyses of randomized trials: One looked at mediating mechanisms, finding that the effects of one internet intervention (Drinker's Check-up) were largely mediated by perceived descriptive norms of alcohol consumption (37) and a subsequent study finding that these mediations were moderated by gender (38). Another study looked at determinants of participation, finding that those with higher alcohol consumption at baseline were more likely to participate in the study (33). As already mentioned, one study looked at cost-effectiveness, finding that the guided intervention was more cost-effective than the unguided intervention (23).

### 3.7. Synthesis of findings

When synthesizing our results, we identified two main differences in study design in the RCTs, that may bear consequences for interpretation of results. Below, we formulate these differences as questions (see Table 2).

**Table 2:** Study design aspects of the 10 RCTs.

**References**	**Could all employees participate in the study, regardless of alcohol use risk level?**	**Was the alcohol focus of the intervention explicit to participants?**
Araki et al. (19)	No (cut-off: GTP)	Yes
Billings et al. (20)	Yes	No
Boss et al. (21)	No (cut-off: drinks in preceding week)	Yes
Brendryen et al. (22)	No (cut-off: AUDIT score)	Yes
Doumas and Hannah (25)	Yes	Yes
Hamamura et al. (26)	Yes	No
Khadjesari et al. (27)	No (cut-off: AUDIT score)	No
Kouwenhoven-Paasmoiij et. (29)	Yes	No alcohol focus
Matano et al. (31)	Yes	No
Pemberton et al. (34)	Yes	Yes
Solenhill et al. (35)	Yes	No alcohol focus

#### 3.7.1. Could all employees participate in the study, regardless of alcohol use risk level?

In six RCTs and one non-randomized trial, all employees were welcome to participate in the study regardless of their level of alcohol use (20, 25, 26, 29, 31, 32, 35) while in four studies only those reporting alcohol problems were able to participate. In these latter studies, inclusion was based on cut-off scores on either AUDIT (22, 27), drinks preceding week (21) or levels of GTP (19). Including everyone in the study regardless of level of alcohol use may introduce a floor effect which could potentially dilute the impact of the intervention, and some researchers tried to handle this risk without explicitly excluding participants. For example, Khadjesari et al. (27) included only those exceeding cut-off scores in the actual study, but everyone was welcome to receive the intervention. In Matano et al. (31), although both low- and medium-risk participants were included and given access to the intervention, participants were categorized according to their alcohol use risk level and then analyzed separately, even though this strategy led to greatly reduced power.

#### 3.7.2. Was the alcohol focus of the intervention explicit to participants?

In some studies, participants were explicitly informed about the alcohol focus of the intervention, while in other studies the focus on alcohol was intentionally masked. This was done, for example, by framing the intervention as how to deal with stress (20, 26, 31) or by having the intervention focus on several different health behaviors, even though the researchers were specifically interested in alcohol (27). As mentioned, some studies focused on health behaviors, with equal weight given to alcohol, physical activity, diet etc (29, 33, 35).

#### 3.7.3. Difficulties in recruitment and follow-up

Recruitment problems were reported in several studies (21, 22, 31). Brendryen et al. (22) discussed these difficulties most extensively; not all employees appeared to use their email account, and the mailing list used by some organizations had not been updated for a long time. Further, a significant amount of time passed between the researchers' first contact with an organization and invitations being sent out to the employees; due to the unexpectedly slow process, recruitment had to be stopped before reaching the number of participants needed. The researchers also noted concerns in organizations about participating in the project related to the randomization aspect of the trial, and about employees spending too much time with the intervention when at work. Matano et al. (31) also encountered recruitment challenges regarding those with more severe problems, citing confidentiality issues as a likely explanation for the difficulty in recruiting this group. In some studies, recruitment issues were discussed in terms of how large a percentage of the organization workforce that participated in the study. In a feasibility study specifically assessing eligibility, recruitment, and attrition, Khadjesari et al. (27, 28) reported that the researchers reached between 4 and 8% of the total workforce in the six organizations with their intervention (PNF for health behaviors), which was considered low when compared to other feasibility studies of brief interventions. Several studies also reported low follow-up rates. For example, Pemberton et al. (34) reported that 35% participated in the 1-month follow-up and 17% participated in the 6-month follow-up, although analyses of missing data suggested that this did not bias the (largely positive) results; Colkesen et al. (24) reported that only 28% participated in the 4-week follow-up.

## 4. Discussion

### 4.1. Summary of findings

In this scoping review, we identified 20 studies about alcohol internet interventions in the workplace. Half of the studies were randomized controlled trials, five were secondary analyses, three were feasibility studies, one was a cohort study and one described the rationale and development of an intervention. No qualitative studies were identified. The interventions consisted of personalized normative feedback and/or cognitive behavior therapy. The gender distribution among participants was similar in most studies and the average age was similar to other alcohol internet intervention studies (16). Organizations where studies were conducted were sparsely described, and the variety of sectors included in the studies (i.e., technology, service, transport, military, and health care) prevent conclusions regarding whether these interventions might be particularly beneficial in certain sectors. All studies except one used only self-report measures to report alcohol-related outcomes and only two studies used work-related outcomes. Effects on alcohol-related outcomes in randomized trials tended to be present when interventions were compared to waitlists but when more intensive interventions were compared to less intensive ones, differential effects were rare. Two design aspects that differed among the studies were identified; one related to inclusion criteria (i.e., some studies included all employees while others used an alcohol-related cut-off) and one related to the framing of the intervention (i.e., some studies were explicit about the alcohol focus of the intervention while others were framed as targeting stress or general health). Several studies reported major difficulties regarding both recruitment and attrition.

### 4.2. What is needed for the field to advance?

The field of alcohol internet interventions is small and the trials conducted to date have evidently encountered many challenges. Improved methodological stringency paired with an awareness of the unique challenges this context provides is needed. Below, we discuss some targeted efforts that the field would benefit from at this stage.

First, the education level of those participating was not reported in most studies. This should be studied in greater detail, especially considering the fact that some research suggests that education level is negatively associated with outcomes in alcohol internet interventions (16). We therefore encourage researchers to collect data on participants' education level and profession. Second, although all RCTs used some form of alcohol-related outcome, specific outcomes in the studies varied (i.e., drinks preceding week, drinking pattern in the preceding month, number of binge episodes, readiness to change etc), complicating comparisons of effects across studies. A consensus statement regarding standardization of outcomes would facilitate future systematic reviews or meta-analyses. Third, only two studies reported on work-related outcomes. Future trials should investigate such outcomes further, for example work productivity and effort-reward imbalance (41). Fourth, as several studies reported major difficulties regarding recruitment, we recommend qualitative studies in order to better understand barriers and facilitators (42). It is still not well understood how these interventions are best implemented within the workplace setting (43), and researchers should prepare meticulously to prevent setbacks related to recruitment and attrition. Fifth, wait lists were common in these trials but there are limitations to using these as they may lead to treatment outcomes being overestimated (16). Active controls should therefore be considered to a greater extent. Sixth, researchers need to be consciously aware of pros and cons of design decisions. For example, if no alcohol-related inclusion criteria is used, the participation pool will be greater which in turn may facilitate recruitment. This may, however, dilute the treatment effect as some participants will not present with alcohol problems and so will not be able to improve. Similarly, framing the focus of an alcohol internet intervention as being about stress or general health may facilitate recruitment, attracting some with alcohol problems who are in denial or who may not have realized that they have an alcohol problem, However, some with alcohol problems but without for example stress issues might neglect an opportunity to participate in such an intervention, as they may feel that this other focus does not apply to them. Future studies could potentially investigate whether this later assumption holds true by randomizing participants to interventions framed in different ways, comparing demographic and clinical characteristics. However, there are also obvious ethical dilemmas in using deception. Lastly, all studies in this review were conducted in high-income countries. As low-and middle income countries tend to have less accessibility to substance use services in general (44, 45), alcohol internet interventions in the workplace could potentially have even more significance in these countries. We therefore strongly encourage research conducted in low- and middle income countries.

### 4.3. Summary of recommendations for future research

1. Detailed information about demographic characteristics such as education level and profession, should be collected, as this might aid in understanding which participants benefit most from these interventions.
2. In order to facilitate comparisons of effects across studies, researchers should include the most common alcohol-related outcome measures in the field.
3. Work-related outcomes should be included to a greater extent.
4. Qualitative studies would likely help in understanding how studies could be designed to decrease problems in recruitment.
5. Researchers should be conscious of how design decisions regarding inclusion criteria and framing of the intervention may influence what conclusions can be drawn.
6. Future studies should include active controls rather than wait list controls.
7. Studies conducted in low- and middle-income countries are sorely needed.

### 4.4. Limitations

This review made use of a broad search strategy and we conducted searches in three widely used databases. Although these databases are large, it is possible that some studies were not identified in this process. Also, we did not search for gray literature. Scoping reviews generally are limited in what can be generalized, in comparison to systematic reviews.

## 5. Conclusions

Alcohol internet interventions hold promise in delivering alcohol prevention in the workplace. However, heterogeneity in study design and difficulties in recruitment complicate interpretation of findings. The field would benefit from a consensus statement on issues related to study design and outcomes.

## Data availability statement

The original contributions presented in the study are included in the article/Supplementary material, further inquiries can be directed to the corresponding author.

## Author contributions

CS: conceptualization, data curation, formal analysis, funding acquisition, investigation, methodology, project administration, writing—original draft, and writing—review and editing. DF: conceptualization, data curation, formal analysis, funding acquisition, investigation, methodology, project administration, and writing—review and editing. AB: funding acquisition, investigation, and writing—review and editing-equal. ZK: conceptualization, formal analysis, investigation, methodology, and writing—review and editing. KS: conceptualization, formal analysis, funding acquisition, investigation, methodology, project administration, writing—original draft, and writing—review and editing. All authors contributed to the article and approved the submitted version.
