# Supplementary material for: What do we know about alcohol internet interventions aimed at employees?—A scoping review
Source: Front Public Health. 2023 Jan 26;11:929782. doi: 10.3389/fpubh.2023.929782 (PMC9909699; doi:10.3389/fpubh.2023.929782)
Supplement: Supplementary file 1 [file Table_1.DOCX]

**Workplace internet alcohol interventions – a scoping review**

Search terms

| population |  |  |  | outcome |  | intervention |
| --- | --- | --- | --- | --- | --- | --- |
| (work*  OR | AND | (digital  OR | AND | (alcohol  OR | AND | (treatment  OR |
| corporate |  | Mhealth |  | drinking) |  | intervention |
| white-collar |  | web* |  |  |  | Cognitive behavio* therapy |
| professional |  | Internet |  |  |  | CBT) |
| labour/labor |  | ehealth/ e-health |  |  |  |  |
| industrial |  | App |  |  |  |  |
| staff |  | Compute* |  |  |  |  |
| organization/ organisation |  | Smartphone |  |  |  |  |
| employ*) |  | Cyber therapy |  |  |  |  |
|  |  | e-intervention |  |  |  |  |
|  |  | e-mental health |  |  |  |  |
|  |  | e-therapy |  |  |  |  |
|  |  | Tele-therapy |  |  |  |  |
|  |  | telehealth |  |  |  |  |
|  |  | online) |  |  |  |  |

**PsycINFO**

(((work* or corporate or white-collar or professional or labor or labour or industrial or staff or organization or organisation or employ*) **and** (CBT or cognitive behavio* therapy or treatment or brief intervention or intervention) **and** (Internet or web or online or compute* or cybertherapy or digital or e-intervention or e-mental health or e-therapy or eHealth or e-Health or tele-therapy or telehealth or app) **and** (drinking or alcohol))).ab,kw,ti.

**Web of Science Search**

TS= (((work* OR corporate OR white-collar OR professional OR labor OR labour OR industrial OR staff OR organization OR organisation OR employ*) **AND** (CBT OR cognitive behavio* therapy OR treatment OR brief intervention OR intervention) **AND** (Internet OR web OR online OR compute* OR cybertherapy OR digital OR e-intervention OR e-mental health OR e-therapy OR eHealth OR e-Health OR tele-therapy OR telehealth OR app) **AND** (drinking OR alcohol)))

**PubMed Search**

((((work*[Title/Abstract]) OR corporate [Title/Abstract]) OR white-collar [Title/Abstract]) OR professional [Title/Abstract]) OR labor[Title/Abstract]) OR labour [Title/Abstract]) OR industrial [Title/Abstract]) OR staff [Title/Abstract]) OR organization [Title/Abstract]) OR organisation OR employ* AND CBT [Title/Abstract] OR cognitive behavio* therapy[Title/Abstract]) OR treatment [Title/Abstract]) OR brief intervention [Title/Abstract]) OR intervention [Title/Abstract]) AND (Internet[Title/Abstract] OR web[Title/Abstract] OR online[Title/Abstract] OR [Title/Abstract] OR compute*[Title/Abstract] OR cybertherapy[Title/Abstract] OR digital[Title/Abstract] OR e-intervention[Title/Abstract] OR e-mental health[Title/Abstract] OR e-therapy[Title/Abstract] OR eHealth[Title/Abstract] OR e-Health[Title/Abstract] OR tele-therapy[Title/Abstract] OR telehealth[Title/Abstract] OR app[Title/Abstract]) AND (drinking[Title/Abstract] OR alcohol [Title/Abstract] AND Journal Article[ptyp] AND ( "2000/01/01"[PDat] : "2021/03/31"[PDat] ) AND English[lang])
